# Effect of Osteopathic Visceral Manipulation on Pain, Cervical Range of Motion, and Upper Trapezius Muscle Activity in Patients with Chronic Nonspecific Neck Pain and Functional Dyspepsia: A Randomized, Double-Blind, Placebo-Controlled Pilot Study

**DOI:** 10.1155/2018/4929271

**Published:** 2018-11-11

**Authors:** Andréia Cristina de Oliveira Silva, Daniela Aparecida Biasotto-Gonzalez, Fábio Henrique Monteiro Oliveira, Adriano Oliveira Andrade, Cid André Fidelis de Paula Gomes, Fernanda de Córdoba Lanza, César Ferreira Amorim, Fabiano Politti

**Affiliations:** ^1^Postgraduate Program in Rehabilitation Sciences, Physical Therapy Departament, Universidade Nove de Julho, UNINOVE, Brazil; ^2^Faculty of Electrical Engineering, Postgraduate Program in Electrical and Biomedical Engineering, Centre for Innovation and Technology Assessment in Health, Federal University of Uberlândia, (UFU), Brazil; ^3^Physical Therapy Program, Universidade Cidade de São Paulo (UNICID), São Paulo-SP, Brazil

## Abstract

Previous studies have reported that visceral disturbances can lead to increased musculoskeletal tension and pain in structures innervated from the corresponding spinal level through viscerosomatic reflexes. We designed a pilot randomised placebo-controlled study using placebo visceral manipulation as the control to evaluate the effect of osteopathic visceral manipulation (OVM) of the stomach and liver on pain, cervical mobility, and electromyographic activity of the upper trapezius (UT) muscle in individuals with nonspecific neck pain (NS-NP) and functional dyspepsia. Twenty-eight NS-NP patients were randomly assigned into two groups: treated with OVM (OVMG;* n *= 14) and treated with placebo visceral manipulation (PVMG;* n *= 14). The effects were evaluated immediately and 7 days after treatment through pain, cervical range, and electromyographic activity of the UT muscle. Significant effects were confirmed immediately after treatment (OVMG and PVMG) for numeric rating scale scores (*p* < 0.001) and pain area (*p* < 0.001). Significant increases in EMG amplitude were identified immediately and 7 days after treatment for the OVMG (*p* < 0.001). No differences were identified between the OVMG and the PVMG for cervical range of motion (*p* > 0.05). This study demonstrated that a single visceral mobilisation session for the stomach and liver reduces cervical pain and increases the amplitude of the EMG signal of the UT muscle immediately and 7 days after treatment in patients with nonspecific neck pain and functional dyspepsia.

## 1. Introduction

Nonspecific neck pain (NS-NP) is a musculoskeletal disorder characterised by pain in the structures located between the superior nuchal line and the spinous process of the first thoracic vertebra [1], which is not associated with a particular disease or modification of anatomical structures [2]. This little-known dysfunction is thought to have a multifactorial cause [2] and contributes to substantial health care costs, work absenteeism and loss of productivity at all levels [2–4].

The specific diagnosis of NS-NP is not clear in the literature or in clinical practice, as several different therapeutic modalities (manual therapy [5], therapeutic exercise [6], auricular acupuncture [7], and acupotomy therapy [8]) have been described as a form of treatment for NS-NP. Furthermore, as the clinical responses from these therapeutic approaches vary in the literature, a specific intervention cannot be identified as a more effective treatment for NS-NP patients. Difficulties in diagnosis and the need to find a specific treatment for this disorder reinforce the need to investigate the possible mechanisms that give rise to cervical pain [9].

One mechanism that remains poorly understood is related to the possibility that visceral disturbances can lead to increased muscle tension and decreased pain threshold in structures innervated at the corresponding spinal level through viscerosomatic reflexes [10]. Sensory nerves enter the spinal cord, and those destined to terminate locally end in the grey matter of the spinal cord where they produce local segmental responses such as excitation, facilitation and reflex actions. In this way, a sensory stimulus may directly affect a motor or sympathetic nerve, or do so through an intermediary interneuron. These interneurons may be either excitatory or inhibitory [10–12]. Therefore, the ongoing afferent stimulation produced from restriction of the mobility of tissues innervated by the phrenic nerve (subdiaphragmatic peritoneum, liver capsule, coronary, and falsiform ligaments) [13, 14] could promote facilitation (irritability) of the internuncial neurons at the levels at which their neural roots are found (between C3 and C5 [13]). This results in increased trapezius muscle tension, as this muscle is innervated by nerve fibres originating from the same medullary segment (C3 and C4).

Another possible visceral influence in the cervical region is the anatomical relationship between the accessory nerve, which innervates the sternocleidomastoid and trapezius fibres, and the vagus nerve, responsible for the parasympathetic control of most abdominal viscera [13]. The accessory nerve has a medullary origin, and arises from neurones of the upper spinal cord, specifically C1-C5/C6. This nerve traverses the posterior cranial fossa to reach the jugular foramen to anastomose with the vagus nerve in its superior ganglion [15].

If nociceptive excitations caused by changes in the functioning and/or visceral mobility also contribute to the emergence of NS-NP, inhibition of the afferent input provided by these alterations could be associated with clinical improvement in individuals with this dysfunction. This inhibition or nociceptive stimulation of visceral origin can potentially be produced by external mechanical action on the viscera through manual manipulation of these structures [16, 17].

The rationale for the use of osteopathic visceral manipulation (OVM) techniques is to improve the mobility [17] and function [18, 19] of the viscera by altering their movement, thereby reducing the excessive afferent input at the spinal level. This could theoretically contribute to normalisation of the excitability state of the afferent neurons of the central nervous system [11].

These neurofunctional relationships and the effects of OVM are currently unclear. Therefore, considering the possibility that viscerosomatic reflexes may be found in patients with NS-NP who exhibit dyspepsia (chronic stomach pain or discomfort with no gastric alteration to explain the symptoms) [20], we tested the hypothesis that possible nociceptive inhibition provided by OVM (stomach and liver) may improve the clinical condition of patients with NS-NP associated with functional dyspepsia.

This pilot randomised placebo-controlled study was designed to evaluate the effect of OVM (stomach and liver) on pain, cervical mobility, and electromyographic activity of the upper trapezius (UT) muscle in individuals with NS-NP and dyspepsia.

## 2. Methods

The present study was a double-blind, placebo-controlled trial with balanced randomization (1:1), approved by the Ethics Committee of the University Nove de Julho (process n°: 02290412.0.0000.5511) and registered in Clinical Trials (NCT03043625). All subjects were informed about the procedures of the study and signed a consent form before any procedure.

### 2.1. Subjects

A convenience sample of 28 patients with NS-NP and dyspepsia participated in the study. Criteria for inclusion were neck pain for at least three months, Numeric Pain Rating Scale (NPRS) [21] between 3 and 8, Neck Disability Index (NDI) [22] between 10 and 24, and the presence of symptoms related to functional dyspepsia, according to the diagnostic criteria of Rome III [20]. The exclusion criteria were presence of structural alterations or cervical abnormalities, history of cervical whip-lash type injury; surgery on the neck, shoulders, chest, or abdomen; reporting of structural changes or any disease in the gastrointestinal tract; treatment for neck pain two weeks prior to the study; the use of analgesics, muscle relaxants, and psychotropic and anti-inflammatory drugs in the 5 days prior to intervention.

### 2.2. Randomization and Blinding

Individuals were allocated to different groups based on numbers randomly generated by a randomization site [23]. Numbers were put into opaque envelopes. The treatment group received osteopathic visceral manipulation group (OVMG) and the control group received placebo visceral manipulation (PVMG). Both the investigators and the participants were unaware of the treatment allocation.

Independent evaluators performed the following procedures: Evaluator 1: triage, random draw of treatments to be performed; Evaluator 2: treatment application; Evaluator 3: EMG data collection; Evaluator 4: EMG signal processing and statistical analysis. Evaluators 3 and 4 were blinded in relation to the groups.

### 2.3. Outcome Assessment

NRS scores for pain and pain area after a single session of OVM were considered the primary outcome and cervical range of movement (ROM) and surface electromyographic (sEMG) activity of the upper trapezius muscle as the secondary outcomes of the study.

### 2.4. Sensory Assessment

The NPRS, translated and cross-culturally adapted for the Brazilian population, was used to assess pain intensity (11-point scale; 0: no pain, 10: the worst possible pain imaginable) [21, 24]. Pain area was documented on a body chart. The drawings were subsequently digitized and pain areas were measured using open-source software ImageJ (Version 1.43, National Institutes of Health, Bethesda, Maryland). The reproducibility of the measurements has been verified in a previous study and was considered acceptable as a pain measurement tool in clinical practice and research [25].

### 2.5. Cervical Range of Motion

Cervical ROM (degree) was measured using a fleximeter (Sanny®, São Paulo, Brazil, L-6010), in a standardized sitting position, to remove errors and movement compensation, except for the movements of rotation, in which they had to stay in the supine position. The equipment was fixed by means of a Velcro strap around the head, with the gauge positioned on the lateral side of the head for the flexion-extension movements, in the frontal region of the head for the right and left lateral inclination movements and at the top for the right and left lateral rotation movements. The reproducibility of the measurements has been verified in a previous study that had intra- and interexaminer reliabilities that ranged from moderate to excellent, which proved its potential for use in clinical practice [26].

### 2.6. Electromyography

The sEMG signals were recorded by an acquisition system with 16 channels (Band pass filter: 20-500 Hz, amplifier gain of 1000 time, CRMR <120dB, EMG System do Brasil Ltda. ®). Two channels were set for the use of the force transducer. The data were recorded with a sample frequency of 2000 Hz and digitalized using analog-digital (A/D) conversion plates, with a 16-bit resolution.

A linear electrode array composed of 10 silver bar electrodes distributed in two columns (5 mm long, 1 mm diameter, and 5 mm interelectrode distance in both directions) was positioned on the UT muscle, 2 cm lateral to the medium point of the line traced between the posterior edge of the acromion and the seventh cervical vertebra [27]. A gel conductor was used to decrease the impedance of the skin. For sEMG signal capture, the skin on the belly of the UT muscle was previously prepared with 70% alcohol to eliminate fatty residues. A ground electrode was placed at the wrist.

### 2.7. Osteopathic Visceral Manipulation

**Figure 1:**
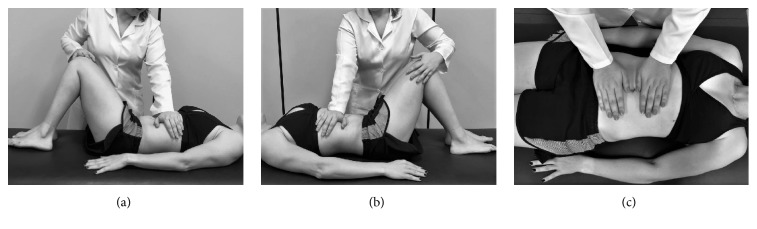
Visceral manipulation techniques for stomach (a), liver (b), and placebo technique (c).

Subjects in the osteopathic visceral manipulation group (OVMG) were submitted to treatment with a single intervention, which involved application of a manipulation technique to the stomach followed by the liver. After an initial evaluation, each participant was instructed to lie down comfortably on an examination table in the supine position, with their lower limbs flexed and abdomen exposed. The therapist was positioned to the right side of the patient. The therapeutic intervention began with the therapist's left hand in contact with the lower region of the stomach. The therapist applied force to this region to move the organ in an upper and lateral left direction while their right hand controlled and directed the subject's knees to the right side, until the therapist noticed an increase in tension in the stomach region (Figure 1(a)). The same procedure was followed for the liver manipulation, but the hand position of the therapist was reversed, with contact in the right epigastric region and the patient's knees directed to the left side. The position was maintained for each organ until the therapist felt a decrease in the tension of the viscera (Figure 1(b)). The mean treatment time was 5 minutes.

Subjects in the placebo visceral manipulation group (PVMG) were treated with a single intervention involving a placebo mobilisation technique, as described by McSweeney [16]. After an initial evaluation, each participant was instructed to lie down comfortably on an examination table in the supine position with their lower limbs extended. The therapist placed their hands over the umbilical region for 1 minute, with no tissue movement (Figure 1(c)).

### 2.8. Procedure

**Figure 2:**
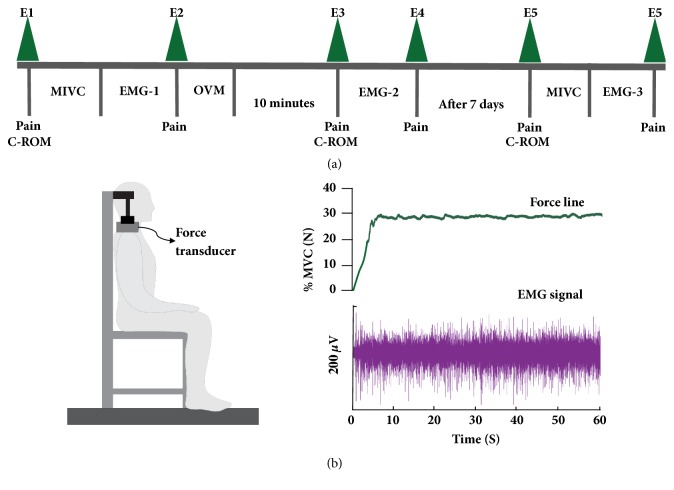
(a) Flow sequence diagram of data recording. (b) Force levels in percentage of the maximum voluntary contraction (MVC). E: evaluation. C-ROM: cervical range of motion. EMG: electromyography. OVM: osteopathic visceral manipulation.

The sequence of experimental events is summarized in Figure 2. The sEMG signal collections were performed in a chair previously instrumented with two force transducers, positioned on the acromion region and adjusted according to the height of each volunteer. The force signals obtained by the transducers were collected, together with the sEMG signal, by the same signal acquisition system. For data collection, volunteers were instructed to sit in the chair with the shoulder and upper limb bare, spine erect, knees at 90° flexion, and feet slightly apart. After the patients were positioned, measurements of pain (NPRS and pain area) and cervical ROM were collected at baseline (E1).

After electrode fixation in the UT muscle that presented greater area of pain, the subjects were instructed to perform three shoulder elevations in maximal isometric voluntary contraction (MIVC) against the resistance of the force transducers for 5 s during verbal encouragement, with an interval of 1 minute between collections. The maximum peak force between force collections (Newtons) was considered as 100% of MIVC. A 30% MIVC training line was established as feedback on the computer screen and subjects were instructed to maintain shoulder elevation over this training line for 60s (EMG-1). After 1 minute rest interval, data on pain was collected (E2). Subsequently, treatment with visceral mobilization or placebo was started. After a ten minute rest interval, new evaluations of NPRS and cervical ROM were performed (E3), followed by a new sEMG signal collection (EMG-2) in the same manner as performed during EMG-1 and data collected on pain after 1 minute rest interval (E4). After a period of 7 days, a further evaluation of pain and cervical ROM (E5) was performed, followed by sEMG signal collection as performed during EMG-1 (3 MIVC initially and shoulder elevation over the training line with 30% MVIC for 60s) (EMG-3). After 1 minute rest interval, data on pain was collected (E6). All participants received training prior to shoulder elevations based on the previously determined force levels.

### 2.9. EMG Signal Processing

The data were analyzed offline using specific routines carried out in the Mathlab program (version R2010a; the MathWorks Inc., Natick, MA, USA).

The amplitude of sEMG was defined as the RMS (root mean square) value of the sig-placebo manipulation on UT muscle activity was verified by the overall RMS value (gRMS) obtained from the mean RMS of the eight channels, since averaging across multiple electrodes increases the stability of the RMS estimates [28].

Muscle fiber conduction velocity (MFCV) was calculated for each force level, using a cross-correlation based algorithm that calculated the time delay corresponding to the maximum of the cross-correlation function, using its time derivative [29].

### 2.10. Data Analysis

The Shapiro-Wilk test was used to test the normality of the data distribution. Data in relation to pain area were log-transformed prior to analyses to negate the effects of heteroscedasticity. Mean age, body mass index and height were compared between groups using independent-sample* t *tests.

The two-way repeated-measures analysis of variance (ANOVA) design was used to analyze the influence of OVM treatment on the pain considering with factors: treatment (OVM vs. PVM) and intervention (pre- vs. immediate posttreatment vs. 7 days after termination of OVM). Specific differences were determined based on post hoc analysis, using Bonferroni correction. The significance level was* p* < 0.05. The data were analyzed using the StatSoft software SPSS 20.0 (SPSS Inc., Chicago, USA).

## 3. Results

Anthropometric data (age, weight, and height) and clinical characteristics assessed by NDI did not differ between the groups treated with visceral manipulation (OVMG) and placebo manipulation (PVMG) (Table 1).

Table 2 shows the results obtained in the evaluations performed preintervention (T1), immediately postintervention (T2) and after 7 days (T3) for individuals treated with visceral manipulation and placebo manipulation.

### 3.1. Pain Analysis

For NPRS, we considered the mean of the data obtained in the evaluations in E1 and E2 as pretreatment values, the mean values of the E3 and E4 evaluations as immediate posttreatment, and the mean E5 and E6 values as post-7 days values.

Significant interaction (treatment vs. groups: ANOVA test) was identified between groups after the treatment for NPRS scores (F = 6.95;* p*< 0.004, *η*_p_^2^ = 0.21) and the pain area (F= 5.35;* p*> 0.008, *η*_p_^2^ = 0.17) (Table 2).

In intra group analysis (post hoc test), significant effects were confirmed for the data collected immediately after treatment in both groups to NPRS (OVMG:* p* <0.001; PVMG:* p* <0.001) and pain area (OVMG:* p* <0.001; PVMG:* p* <0.001) (Table 2). For the data collected after 7 days of treatment, only OVMG presented statistically significant differences for NPRS (*p* <0.001) and pain area (*p* <0.001) in relation to data collected preintervention (baseline).

### 3.2. Cervical Range of Motion

No significant interaction (treatment vs. groups: ANOVA test) was identified between groups after the treatment for cervical ROM. In intra-group analysis, only OVMG presented a significant increase of the cervical ROM for extension and right side flexion movements after immediate treatment (*p* <0.001) and left side flexion after 7 days of treatment (*p* <0.001) (Table 2).

### 3.3. Electromyography

**Figure 3:**
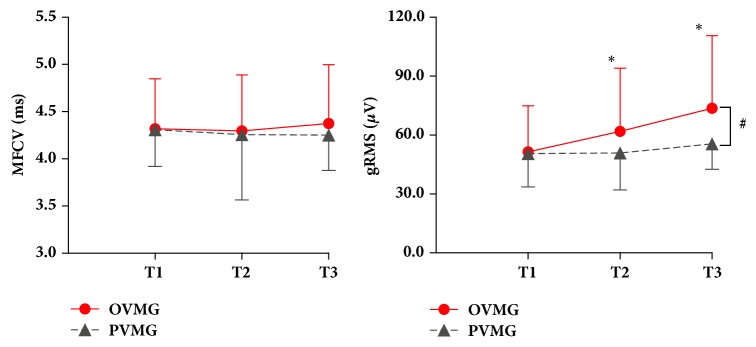
Mean and standard deviation of the muscle fiber conduction velocity (MFCV) and electromyographic amplitude (RMSg) of the upper trapezius muscle recorded pretreatment (T1) immediate posttreatment (T2) and 7 days after treatment (T3) with osteopathic visceral manipulation (OVMG) or placebo visceral manipulation (PVMG). The data were obtained with 30% of the maximum voluntary contraction from the shoulder elevation.*∗* Significant difference in relation to T1. # Significant difference between group.

Figure 3 shows the mean values (SD) of muscle fiber conduction velocity (MFCV) and electromyographic amplitude (gRMS), before, immediately after, and 7 days after treatment, for VMG and PMG. The analysis of the influence of the treatment with visceral manipulation in the MFVC revealed no significant differences for the treatment interactions (F=0.06,* p*=0.94; *η*_p_^2^=0.002) and treatment vs. group (F=0.11,* p*=0.89; *η*_p_^2^=0.004). However, significant differences were observed in the EMG signal amplitude analysis for treatment interactions (F=18.80,* p*<0.001; *η*_p_^2^=0.42) and treatment vs. group (F=6.76,* p*=0.002; *η*_p_^2^=0.20).

## 4. Discussion

The results of this study verified our hypothesis that possible nociceptive inhibition provided by OVM of the stomach and liver reflects an improvement in the clinical status of patients with NS-NP associated with dyspepsia. The significant decrease in pain, measured by NPRS and the area of pain, together with a significant increase in the amplitude of the EMG signal of the UT muscle in the OVMG immediately after the intervention and after 7 days suggests that viscerosomatic reflexes may be present in NS-NP patients with dyspepsia. The mechanisms underlying this reflex are not yet understood and require further investigation; however, these results strengthen the possibility that OVM of the stomach and liver could contribute to the treatment of these patients.

There was a significant decrease in pain symptoms immediately after the intervention for the groups treated with OVM and the placebo. Similar results have been reported for patients with NS-NP who were treated with acupuncture for pain control [30], as well as in those with musculoskeletal disorders such as fibromyalgia [31] and acute and chronic lower back pain [32]. One possible explanation for these results is that sensory stimulation by touching the skin activates mechanoreceptors in the skin that convey light touch and activate A*β* afferents fibres, thereby inhibiting pain [33]. In addition, nonspecific factors such as contact time, expectation and the ritual related to the therapeutic approach may have also led to the observed reduction in pain immediately after performing interventions with OVM and the placebo [34].

In relation to EMG of UT muscle activity, previous studies have found that pain promotes a decrease in the electromyographic activity of this muscle during isometric contraction [35, 36]. Thus, the increase in signal amplitude (RMSg) found only for the OVMG in this study suggests a possible effect on contraction of this muscle promoted by OVM of the liver and stomach. The fact that no changes were found in the MFCV for both groups could be related to the level of force needed during the evaluation, as the MFCV appears to remain constant during sustained isometric exercises at relatively low contraction levels (10–30% MVC) [28, 37, 38].

The results observed for the cervical ROM in this study did not contribute to a better understanding of the physiological mechanisms of OVM. The differences observed in the OVMG post-intervention were heterogeneous, with significant differences in the movements of extension and right-side flexion immediately after OVM and for left-side flexion 7 days after the intervention. There was no significant improvement in the PVMG, so we are unable to conclude that these differences are related to treatment with OVM. Thus, these results must be interpreted with caution.

Although explanation of these findings is not straightforward, the responses observed for pain and EMG activity of the UT muscle after OVM indicate that the visceral stimulus provided by the manipulation techniques applied in this study may be related to some physiological mechanism (not yet reported clearly in the literature) that inhibited pain and muscle activity. This physiological effect could be due to muscle relaxation and a consequent clinical improvement, evidenced by the decrease in pain reported by individuals in the OVMG 7 days after treatment. These observations reinforce our initial hypothesis that visceral changes can produce a nociceptive input that can promote alterations in the muscular activation threshold at the spinal level and, consequently, changes in the activation pattern of the muscles corresponding to the affected spinal level as previously suggested [11, 16, 39].

The results of this study reinforce the possibility that spinal facilitation of the internuncial neurons occurred in the OVMG at the level of the neural roots of the phrenic nerve (C3-C5) that innervates the diaphragm muscle, the subdiaphragmatic peritoneum [13], coronary ligaments, sickle cell, and liver capsule [14]. This is supported by previous studies which reported the presence of trophic changes in the superficial and deep paraspinal muscles in patients with gallbladder dysfunction [39], an increase in the pressure pain threshold of the paraspinal muscles of L1 after manipulation of the sigmoid colon [16], and decreased mobility of the right kidney and bladder in patients with nonspecific lower back pain [17].

The results of this study can be considered promising for a better understanding of mechanisms involving viscerosomatic reflexes; however, they should be interpreted with caution given the important methodological limitations of the current study. These include the lack of calculation of sample size ratio (although mitigated by the effect size calculation), presentation of the effects observed after only a single treatment session, and absence of prior evaluation of visceral mobility, which is usually performed subjectively by the therapist, which makes scientific reproduction difficult. Another limitation was that we did not assess clinical variables related to fibromyalgia (visceral pain, headache, sleep, and mood disorders), which is a common comorbidity in these patients [40]. This has important implications regarding the clinical management of patients with overlapping chronic pain [41], and our focus on only two pain condition (NS-NP and dyspepsia), both in the context of diagnosis and treatment, may be an important limiting factor in relation to our understanding of the results observed after OVM.

To our knowledge, there has been no randomised controlled trial assessing the effectiveness of OVM as a complementary therapy for the relief of acute pain or for improving cervical function in NS-NP patients. Therefore, the present study provides the basis for future studies to assess the efficiency of treating NS-NP with OVM, as previously suggested [10, 14].

## 5. Conclusions

The results of this pilot study indicate that a single session of osteopathic visceral manipulation for the stomach and liver reduces cervical pain and increases the amplitude of the upper trapezius muscle EMG signal immediately and 7 days after treatment in patients with nonspecific neck pain and functional dyspepsia. Patients treated with placebo visceral mobilisation reported a significant decrease in pain immediately after treatment. The effect of this intervention on the cervical range of motion was inconclusive. The results of this study suggest that further investigation is necessary.

## Figures and Tables

**Table 1:** Mean and standard deviation of demographic and clinical data.

	**OVMG**	**PMG**	***p *Value** *∗*
**Age (years)**	23.85±6.27	27.01±9.90	0.18
**Weight (Kgf)**	65.35±15.66	63.64±9.90	0.74
**Hight (cm)**	1.62±0.07	1.64±0.07	0.58
**NDI**	15.07±3.55	15.14±2.87	0.95

**OVMG: **osteopathic visceral manipulation group.** PMG: **placebo manipulation group.** NDI:** Neck Des.

*∗* Independent-sample *t* tests.

**Table 2:** Mean and standard deviation (SD) and interactions (ANOVA) of the values of cervical ROM and pain, obtained pretreatment (T1), immediately posttreatment (T2), and 7 days after treatment (T3).

	**Osteopathic Visceral Manipulation Group** **(Mean±SD)**			**Placebo Manipulation Group** **(mean±SD)**			**Anova test interations**			
	**T1**	**T2**	**T3**	**T1**	**T2**	**T3**	**Treatment**		**Treatment vs groups**	
							***p* value**	**Effect size**	***p* value**	**Effect size**
**Cervical ROM (**°**)**										
Extension	50.11±18.10	59.59±19.67*∗*	56.16±18.31	57.05±11.32	57.28±7.25	57.85±8.08	0.03	0.12	0.06	0.10
Flexion	56.69±9.85	57.19±10.33	60.66±7.61	57.88±15.99	59.71±12.12	60.45±13.91	0.07	0.09	0.63	0.01
Right Side Bending	41.45±9.50	47.04±10.14*∗*	44.28±7.29	43.31±8.01	47.00±8.58	47.33±8.27	0.01	0.15	0.62	0.01
Left Side Bending	43.26±12.02	46.40±10.35	48.40±10.10*∗*	46.49±11.21	48.64±11.47	47.73±8.20	0.04	0.11	0.33	0.04
Right Rotation	63.85±10.79	62.97±10.91	61.33±11.07	59.09±15.17	56.80±14.60	60.14±14.63	0.71	0.01	0.34	0.04
Left Rotation	65.66±15.58	64.33±9.55	64.33±14.6359	59.61±15.62	61.16±11.33	61.00±13.66	0.99	<0.001	0.76	0.01

**Pain Analysis**										
NPRS	5.85±1.48	4.39±1.86*∗*†	3.21±2.08*∗*	5.82±1.57	4.50±1.96*∗*	4.71±1.72*∗*	<0.001	0.58	0.004	0.21
Area ^‡^	6.11±0.90	5.50±1.02*∗*†	4.43±2.11*∗*	5.54±0.85	5.00±0.77*∗*	5.05±0.83	<0.001	0.35	0.008	0.17

^‡^ Log-transformed values (arbitrary units).

*∗* Significantly different from T1 (p < 0.001).

† Significantly different from T3 (p < 0.001).

## Data Availability

The datasets generated during and/or analyzed during the current study are available from the corresponding author on reasonable request.
